# Cultural and linguistic adaptation of the multi-dimensional OXCAP-MH for outcome measurement of mental health among people living with HIV/AIDS in Uganda: the Luganda version

**DOI:** 10.1186/s41687-021-00306-0

**Published:** 2021-04-07

**Authors:** Kenneth R. Katumba, Yoko V. Laurence, Patrick Tenywa, Joshua Ssebunnya, Agata Laszewska, Judit Simon, Anna Vassall, Eugene Kinyanda, Giulia Greco

**Affiliations:** 1 MRC/UVRI & LSHTM Uganda Research Unit (Social Aspects of Health), Plot 51-59 Nakiwogo Road, Entebbe, +256 Uganda; 2 Centre for Health Economics in London, (Department of Global Health and Development), London School of Hygiene & Tropical Medicine, London, UK; 3 MRC/UVRI & LSHTM Uganda Research Unit & Senior Wellcome Trust Fellowship (Mental Health Section), Entebbe, Uganda; 4 Butabika National Referral and Teaching Mental Hospital, Kampala, Uganda; 5 Center for Public Health, (Department of Health Economics), Medical University of Vienna, Vienna, Austria; 6 University of Oxford, (Department of Psychiatry and HERC), Oxford, UK

**Keywords:** Capability approach, Mental health, Quality of life, HIV/AIDS, Uganda, PROMs

## Abstract

**Background:**

It is rare to find HIV/AIDS care providers in sub-Saharan Africa routinely providing mental health services, yet 8–30% of the people living with HIV have depression. In an ongoing trial to assess integration of collaborative care of depression into routine HIV services in Uganda, we will assess quality of life using the standard EQ-5D-5L, and the capability-based OxCAP-MH which has never been adapted nor used in a low-income setting. We present the results of the translation and validation process for cultural and linguistic appropriateness of the OxCAP-MH tool for people living with HIV/AIDS and depression in Uganda.

**Methods:**

The translation process used the Concept Elaboration document, the source English version of OxCAP-MH, and the Back-Translation Review template as provided during the user registration process of the OxCAP-MH, and adhered to the Translation and Linguistic Validation process of the OxCAP-MH, which was developed following the international principles of good practice for translation as per the International Society for Pharmacoeconomics and Outcomes Research’s standards.

**Results:**

The final official Luganda version of the OxCAP-MH was obtained following a systematic iterative process, and is equivalent to the English version in content, but key concepts were translated to ensure cultural acceptability, feasibility and comprehension by Luganda-speaking people.

**Conclusion:**

The newly developed Luganda version of the OxCAP-MH can be used both as an alternative or as an addition to health-related quality of life patient-reported outcome measures in research about people living with HIV with comorbid depression, as well as more broadly for mental health research.

## Introduction

Studies in sub-Saharan Africa report depression rates of 8–30% [1–4] among the 25 million people living with HIV [5]. However, the majority of HIV/AIDS care providers in the region do not routinely provide mental health services [1]. In Uganda the call for the integration of mental health and other chronic conditions in HIV care by the National HIV and AIDS strategic plan 2015–2020 [6] has received support in the form of the Ministry of Health (MoH) consolidated guidelines for the prevention and treatment of HIV in Uganda [7]. These guidelines have for the first time called for the assessment and management of depression in people living with HIV [6, 7].

Depression is associated with lower quality of life [8] and a number of negative clinical and behavioural outcomes, including rapid HIV disease progression leading to mortality [9, 10], poor adherence to HIV treatment [3, 11–13], risky sexual behaviour [11, 14–16] and increased utilisation of health facilities [3, 11, 13–15].

The HIV + D study is an on-going cluster randomized controlled trial led by the MRC/UVRI & LSHTM Uganda Research Unit in partnership with the STD/AIDS control programme of the Ugandan ministry of health, that evaluates the effectiveness of integrating stepped, collaborative care of depression into routine HIV services in Uganda. An economic evaluation is planned alongside the trial and it was thus necessary to identify an appropriate outcome measure to use in the cost-effectiveness analysis.

Patient-reported outcome measures (PROMs) have been used to evaluate healthcare interventions via cost-effectiveness analyses [13, 17, 18]. Health-related quality of life (HRQoL) PROMs are a systematic approach for obtaining meaningful, subjective accounts from patients on their quality of life [19] and satisfaction with health services [13, 17, 18]. They can be generic and therefore used across diseases and conditions, such as the 36- and 12-item Short Form Surveys (SF-36, SF-12) [20, 21], and the EQ-5D [22]. The sensitivity and appropriateness of these HRQoL PROMs in more specific populations such as those with comorbidities has however been questioned [13]. The selection of an appropriate HRQoL PROM is therefore dependent on the target population, the nature of the intervention, and its ultimate use, with many trials supplanting a disease-specific HRQoL PROM that will be more sensitive to treatment with a generic HRQoL PROM that is more comparable across populations and disease-conditions [13, 17].

Generic HRQoL PROMs have been used globally [23, 24], while disease-specific HRQoL PROMS have mainly been developed and used in Europe and other high-income countries to assess quality of life in patients with depression [25–28]. No disease-specific HRQoL PROM has however been developed or adapted to assess quality of life in patients with depression in Africa.

Over the years, there has been growing interest in the development and use of PROMs that include a multidimensional understanding of wellbeing. Some of the methodological progress in this area has been grounded in Amartya Sen’s capability approach, which focuses on the freedom that people have to be or to do what that they have reason to value, given their personal characteristics and external circumstances [29, 30].. PROMs that are rooted in the capabilities approach include domains that are relevant and important to the quality of life of people with mental health disorders, such as the quality of their relationships, sense of belonging and acceptance, self-perception, autonomy, freedom of choice, and feeling of hope [31]. In the HIV + D trial [32] in Uganda we proposed the use of the Oxford CAPabilities Questionnaire for Mental Health (OxCAP-MH) alongside the EQ-5D-5L to assess both the broader-dimensional and the generic HRQoL of people living with HIV/AIDS and depression.

The OxCAP-MH is a multidimensional outcome measure for mental health interventions, based on the capabilities approach. It was originally developed in the UK and has been translated and adapted so far for the German, Hungarian and Chinese populations [27]. To date the questionnaire has been psychometrically validated in the UK, Germany and Austria for use among populations with mental health disorders [25, 33, 34]. It has also been used in several trial-based mental health economic evaluations [28, 35].

It comprises 16 items which reflect the 10 central capabilities by Nussbaum [30]: social networks, love and support, suitable accommodation, neighbourhood safety, daily activities, losing sleep, enjoy recreation, potential for assault, discrimination, influence local decisions, freedom of expression, appreciate nature, respect and appreciation, planning one’s life, imagination and creativity, and access to interesting activities [25, 27]. OxCAP-MH offers a multi-faceted and broad scope for assessing the quality of life of people living with HIV/AIDs and depression, as it includes aspects of social integration, discrimination and stigma [25, 27].

Though the OxCAP-MH has only been validated in high-income settings in Europe, it was chosen for the HIV + D study in Uganda to complement the EQ-5D-5L outcome measure as it captures the effects of the trial beyond health.

In this study we present the results of the translation and the validation process for cultural and linguistic appropriateness of the OxCAP-MH tool for use with people living with HIV/AIDS and depression in Uganda.

## Methods

The translation and the cultural validation process was coordinated by the HIV + D Health Economics team, and supported by a team of mental health experts consisting of clinical psychiatrists, clinicians, research assistants and two expert clients. The quality of this process was ensured by adhering to the Translation and Linguistic Validation (TLV) process of the OxCAP-MH as stipulated by the custodian of the tool, which was developed following the international principles of good practice for PROMs’ translation as per the International Society for Pharmacoeconomics and Outcomes Research’s (ISPOR) standards [36]. We also used the Concept Elaboration document, the source English version of OxCAP-MH, and the Back-Translation Review template as provided during the user registration process of the OxCAP-MH [37].

**Fig. 1:**
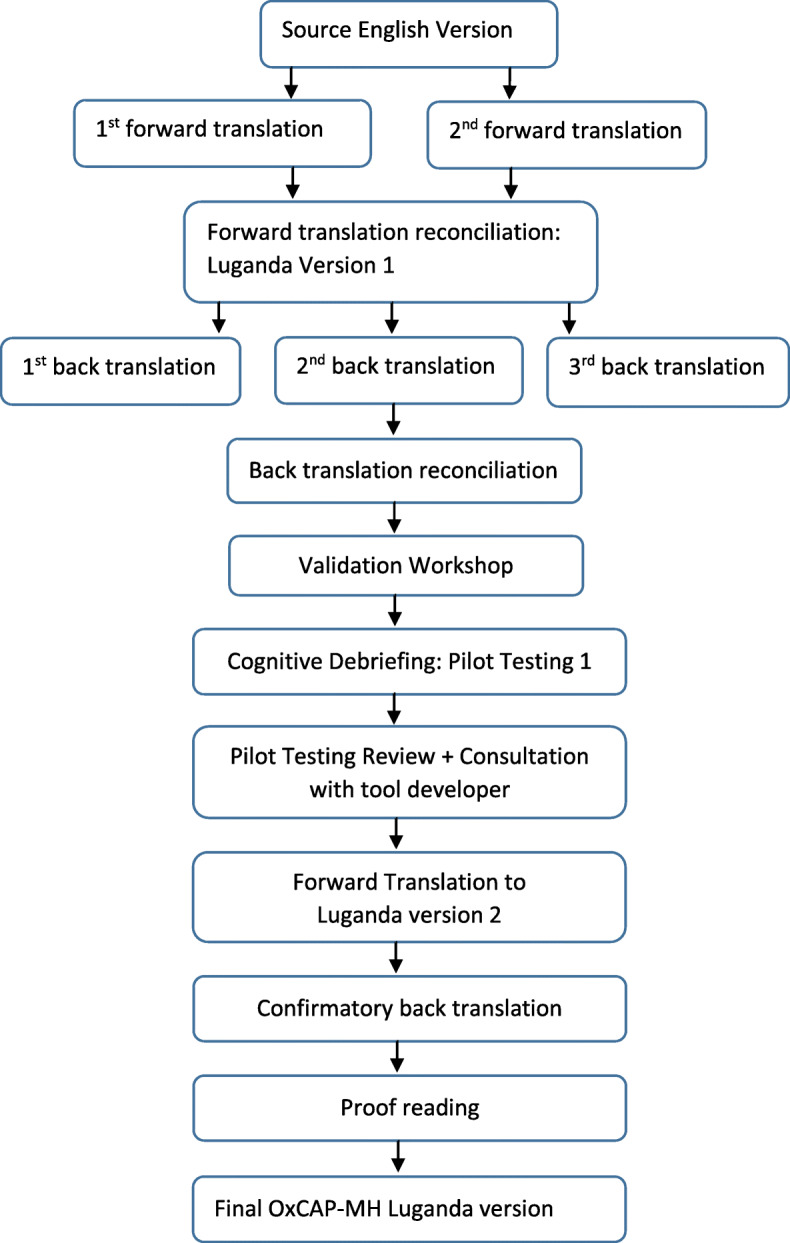
Translation of the Source OxCAP-MH English version into Luganda according to the Translation and Linguistic Validation process

Figure 1 below outlines the process of translating and validating the OxCAP-MH into Luganda.

### Forward translation and forward translation reconciliation

The first forward translation from English to Luganda was based on the source English version of the OxCAP-MH, and two additional questions derived from the earlier developmental version of the English OxCAP-MH from the paper by Simon et al. (2013) [38]. Guided by the concept elaboration document, the forward translations were carried out by two independent translators: a Health Economist and a Clinical Psychologist. Both translators were native Luganda speakers and proficient in English, with extensive experience in medical translations. From these translations, a reconciled Luganda version (OxCAP-MH Luganda version 1) was produced that most accurately represented the concepts within the source English version.

### Back-translation and review

OxCAP-MH Luganda version 1 was sent for blinded back-translation to three independent Luganda speakers with proficiency in English and experience in translations: a social scientist; a research assistant; and a BSc Information Technology student.

A multi-disciplinary team formed by the translators and three additional anthropologist/social scientists was appointed to review specific queries on wording and produce a reconciled version, and supported by the developers.

### Validation workshop

According to the TLV process, the OxCAP-MH Luganda version 1 was ready to be piloted at this stage, but we added a further step in the process to improve the validity of the translation before piloting. We organized a validation workshop for the wider HIV + D trial team, which brought together clinical psychiatrists, health economists, research assistants, expert clients – patients with both HIV/AIDS and depression but acting as peer leaders to the other patients, and District Health Officers (DHOs). The objectives of the workshop were to discuss and validate by team agreement both the forward and backward translations, terminologies and wording; and to address any other considerations specific to people living with HIV/AIDS and depression. Consensus was reached on a second version (OxCAP-MH Luganda version 2). None of the key concepts of the OxCAP-MH was changed during this workshop, but concerns about the local applicability of concepts like ‘*enjoying nature’*, ‘*interesting forms of activity’*, and ‘*freedom to use imagination to express oneself*’ were cited during this workshop.

### Pilot testing (cognitive debriefing) and review

The aim of the pilot testing was to confirm whether the OxCAP-MH Luganda version 2 was accurately understood and acceptable to a sampled population of people living with HIV and depression. We randomly selected 20 participants of the main HIV + D trial pilot from 4 facilities. The participants were contacted by the health facility supervisors in the HIV + D trial by telephone, or via community contacts.

Two strategies were compared for delivering and validating the tool: interviewer-administered (IA), where the interviewer administered the tool to the respondents one at a time, sequentially, capturing the responses on the paper questionnaires; and self-administered (SA) [38], where two interviewers guided a group of respondents through each question, and the respondents wrote their own responses on the paper questionnaires independently. For respondents that could not write, the second interviewer documented their responses. IA and SA questionnaires were identical.

The pilot was carried out after obtaining appropriate institutional and statutory ethical approvals. All study participants gave written informed consent, and those that couldn’t sign used their fingerprint in the presence of a witness.

Findings from the pilot were reviewed by the trial team and by the custodian of the OxCAP-MH. In addition to amendments to the Luganda version, the official English version of the OxCAP-MH was also slightly modified (OxCAP-MH English (UK) 2020) to accommodate the broader international context, e.g. for Luganda-speaking populations.

### Forward and confirmatory back-translation of version 2

Following the pilot, key questions with complex meanings were reworded to fit the local context and to be understood by study participants without altering the original meaning of the questions. This was done by agreement between the HIV + D Health Economics team and the tool’s custodian.

These questions were back translated from Luganda into English by two independent and blinded social science researchers, and the reconciled OxCAP-MH Luganda version 2 was assessed for consistence and flow by the HIV + D Health Economics team.

### Proofreading and review

Using the Concept Elaboration guidance document, the OxCAP-MH Luganda version 2 was proof-read by a co-investigator of the HIV + D trial, who was not involved in any other part of the OxCAP-MH validation process. Review of the feedback created the final OxCAP-MH Luganda version 3 which following a final round of consultation with the tool’s custodian, was approved as the official Luganda version of the OxCAP-MH and is now accessible for further use at https://healtheconomics.meduniwien.ac.at/downloads/oxcap-mh/.

## Results

The official Luganda version of the OxCAP-MH presented in this paper is equivalent to the latest source English version and contains 16 items. Two questions (on home ownership and life expectancy) were not included. The question on life expectancy was kept in the trial patient questionnaire separately from the OxCAP-MH, as this was deemed potentially informative in the context of the main HIV + D trial analysis.

Twenty-nine phrases were translated from the source English questionnaire into Luganda. Of these, 16 were questions, two were additional questions not included in the scoring, and four were instruction phrases. Six were different response options, and one was a sentence to explain the aim of the questionnaire at the beginning.

In following the formal process as shown by Fig. 1, we produced three versions of the Luganda OxCAP-MH, the third version being the final officially accepted version. The first version was created after reconciliation of the two independent forward translations; the second version after the back translation, validation workshop, cognitive debriefing, a second forward translation and confirmatory back translations, and changing seven out of the 29 phrases (24%); and the final version after proofreading.

Three men and 15 women from four health centres participated in the pilot. Even after replacement and follow-up phone calls, we were not able to successfully reach a fifth respondent in two of the health facilities. The mean age of the participants was 43 years (range 23–58). The characteristics of the participants are detailed in Table 1. Each interview session lasted an average of 5–13 min per participant for the interviewer-administered questionnaires and 30 min for the self-administered questionnaires.

**Table 1:** Characteristics of the pilot interview participants

Health Facility	Participant Id	Age	Sex	Interview Strategy	Interview Time	Depression Score	
						PHQ2	PHQ9
Mpigi HCIV	MP01	58	Female	IA	10	5	14
	MP03	53	Female	IA	18	3	11
	MP10	39	Female	IA	8	4	12
	MP15	23	Female	IA	7	3	19
	MP33	49	Female	IA	12	3	10
Butoolo HCIII	BT02	47	Female	SA	33	3	12
	BT07	45	Female	SA	33	6	16
	BT08	26	Female	SA	33	3	18
	BT12	32	Female	SA	33	4	15
	BT29	49	Male	SA	33	4	10
Buwama HCIII	BW08	53	Female	IA	8	5	20
	BW07	38	Male	IA	13	6	18
	BW03	61	Male	IA	14	6	15
	BW17	38	Female	IA	14	6	14
Nkozi Hospital	NZ26	54	Female	SA	13	6	17
	NZ08	43	Female	SA	13	3	10
	NZ01	40	Male	IA	4	4	14
	NZ22	34	Male	IA	13	4	15

**†** PHQ9 and PHQ2 are screening instruments for depression, where: PHQ2 ranges from 0 to 6 and a value of zero indicates no depression, and a value greater than 3 indicates severe depression; PHQ9 ranges from 0 to 27 and a value of 0-4indicates no depression, and a value greater than 14 indicates severe depression

Twelve of the sixteen questions (75%) were easy for the participants to understand. As shown in Table 2 below, the participants found it difficult to understand and answer the initially translated questions on “appreciating nature”, “imagination and creativity”, and “access”. The Translation Coordination Team also expressed concern about potential confusion around the same three concepts, as well as the questions about “daily activities” and “enjoying” recreation. Solutions to these concerns were found after iterative discussions with the custodian of the OxCAP-MH.

**Table 2:** Results of the pilot testing

Item	Content	Concern by participants (Y/N)	Concern by TCT (Y/N)	Details of concern	Solution to concern
Q1	Daily activities	N	Y	Definition of health in Luganda	Included term that acknowledges we mean mental and physical health, not lifestyle
Q2	Social networks	N	N		
Q3	Losing sleep	N	N		
Q4	Enjoying recreation	N	Y	Concept non-existent in traditional local culture. Equivalent translation hard to get	Include an explanation that ‘things that make you happy’ excludes anything with duty, like working or caring activities
Q5	Suitable accommodation	N	N		
Q6	Neighbourhood safety	N	N		
Q7	Potential for assault	N	N		
Q8	Discrimination	N	N	Some respondents raised another reason for discrimination not on the provided list	Added the option ‘others’ to the reasons for discrimination
Q9a	Influencing local decisions	N	N		
Q9b	Freedom of expression	N	N		
Q9c	Appreciating nature	Y	Y	Concept non-existent in traditional local culture. Equivalent translation hard to get	Used locally appropriate examples
Q9d	Respect and appreciation	N	N		
Q9e	Love and support	N	Y	Luganda term for love has various meanings	Interviewer to be trained to use proper intonation of the word
Q9f	Planning one’s life	N	N		
Q9g	Imagination and creativity	Y	Y	Freedom of imagination and creativity not traditionally expressed through talent	Opted for the expression my thoughts, feelings and ideas, then added singing, dancing and talents as examples
Q9h	Access	Y	Y	There was no equivalent of ‘Interesting’ forms of activity/employment	Added locally appropriate examples

At the end of the qualitative analysis of the pilot study results, 11 changes were proposed to the first Luganda version – nine to questions, one to a response option, and one to a particular word. In total, seven of the suggested changes (64%) were agreed and incorporated in the second Luganda version, resulting in a total of six changed phrases and one changed word.

Some of the proposed changes referred to concepts that were culturally inappropriate or non-existent in Luganda, thereby making it hard to get equivalent translations. These concepts included appreciating nature; imagination and creativity; enjoying recreation; and access.

Particularly challenging was the translation of two English words which in Luganda have multiple meanings, with specific meaning being defined by the context: ‘love’ – ‘*okwagala*’, which can be loving, liking, and sex; and ‘health’ – ‘*embera y’obulamu’* that can be ‘lifestyle’, ‘nature of life’ and ‘standard of living’.

For example, the translation of Q9c (*I am able to appreciate and value plants, animals and the world of nature*) was difficult since this concept does not exist in the Kiganda culture. After discussion with the custodian of the OxCAP-MH, it was agreed that in order to maintain integrity and harmonised scoring of the OxCAP-MH, the Luganda translation of the question would be changed from the first forward translation - ‘*Nzisa ekitiibwa era manyi omugaso gw’ebiliime, ebisoolo n’ensi y’obutonde*,’ to ‘*Nnina obusoboozi obusiima ettaka lyaffe, amazzi (nga ogaseko amazzi g’enkuba) ebibiira, n’embela y’obudde,*’ which back translates to “I am able to appreciate our land, water (including rain water) forests and weather.”

Q9g (*I am free to use my imagination and to express myself creatively* e.g. *through art, literature, music,* etc.) was also difficult since the concepts of imagination and creativity are not the same in the Ugandan context compared to a Western/European context, and neither is the option to indicate the existence of freedom of expression. The Translation Coordination Team suggested changing the wording to ‘I am free to express what is in my heart’ and give examples by adding opposing emotions ‘such as when I am happy, when I am sad, when I an angry, when I am afraid, when I am excited.’ After consultation with the custodian of the OxCAP-MH, the wording as ‘I am free to express my thoughts, feelings and ideas (e.g. through singing, dancing, talking to someone, etc.)’ was agreed, to maintain the originally intended content of ‘imagination’ and ‘express myself creatively’.

Q9h (*I have access to interesting forms of activity, or employment*) was unclear for respondents as this concept does not exist in Kiganda culture. We suggested adding examples to the statement to make it culturally accessible. The final agreed text was ‘I am able to participate in meaningful activities (or work), such as church activities, caring for family, hobbies, sports, etc.’

Two proposed changes involved phrases with potentially lost nuances, on the questions of neighbourhood safety and potential for assault. We contemplated if questions 6 (on safety) and 7 (on assault) were mutually exclusive. After consultations with both the experts and the custodian of the tool, we agreed that the two were mutually exclusive with Q6 referring to physical safety in neighbourhood and Q7 to different kinds of assault including safety at home, at work or in school, albeit being very broad in terms of type of violence and people who perpetrate (family, teacher, stranger). We therefore left both as they were.

One proposed change was an additional response option to Q8a on the expected reasons for discrimination. The respondents in the pilot study suggested adding ‘economic status’ as an option because it was the most common reason why they were discriminated against. Following discussions with the custodian of the tool, it was agreed that in order to keep the integrity of the original OxCAP-MH, the current answer options be kept, but an additional option of “*Other*”, proceeded by space for respondents to write their answer, be adopted instead. This proposed change has also been included in the English, German and Hungarian versions (2020).

## Discussion

The final official Luganda version of the OxCAP-MH was obtained following a systematic iterative process of cultural and linguistic validation and adaptation with the involvement of a multidisciplinary team. It is equivalent to the English version in content, but culturally acceptable, feasible and comprehensible by Luganda-speaking people. Although the tool was piloted among people with HIV and depression, it is suitable for use by all Luganda speakers. It is the first translation of the tool in a low-income country.

Our study followed a robust methodological design similar to Simon et al., 2018 [27], which reflects the principles of good practice for translation of PROMs adopted by ISPOR [27, 36]. Three extra steps were added to the recommended process: a validation workshop after the first back translations, which provided expert opinion on the anticipated acceptability and ease of use of the tool in the target population; a second forward translation of the edited phrases; and a confirmatory back translation of the second forward translation.

The differences in the cultural, social and political concepts between the UK where the tool was developed and Uganda were apparent in various questions. For example, in Q9a respondents are asked about their ‘voice’ in influencing local decisions. In Uganda the concept of local participation is not widespread. Other questions needed culturally appropriate examples: Q9c on appreciating nature and Q9g on using one’s imagination. In Q8 a reason for discrimination was added. The cultural differences were pronounced in Q9b (freedom of expression), Q9c (appreciating nature), Q9g (imagination) and Q9h (access) as they proposed concepts that are culturally inexistent in Uganda. Capabilities questionnaires could be more flexible in the use of locally appropriate concepts. An alternative approach would be to develop a bottom-up list of locally relevant capabilities [39].

The tool was administered using two approaches. The interviewer-administered approach caters for both literate and illiterate respondents, despite being longer and more taxing in settings where many participants are involved. The self-administered approach is less repetitive and reduces interviewer fatigue. These advantages and disadvantages are specific for our context but might vary even across African settings. Prospective users should therefore choose and adapt a questionnaire delivery strategy that best fits their context.

Due to the cultural and contextual differences between the original European and the Ugandan setting, it was necessary to undertake an approach with multi-step consultations with the custodian of the tool, and with a team of anthropologists and social scientists. This study has highlighted the importance of following a systematic and standardised step-by-step linguistic and cultural adaptation process when transferring PROM instruments to different settings and languages. PROMs developed in a specific country/language cannot be taken “off-the-shelf” and used in a different setting.

Our study has a number of limitations. We were unable to employ an accredited professional company to translate the source English version to Luganda, however we engaged experienced and knowledgeable researchers. We believe this presents an opportunity, especially in LMICs, to translate and adopt the OxCAP-MH with limited resources, while still maintaining high standards of scientific rigor.

The OxCAP-MH is a capability-based measure that was developed around Nussbaum’s ten central human capabilities. These represent aspects of quality of life that are considered the backbone for a life of human dignity that goes beyond survival and health and includes more complex components of what makes a life worth living, such as dignity, relationship, and discrimination. These core dimensions should be included in any exercise that assesses quality of life and human development across different populations. The Luganda version of the OxCAP-MH will provide an adequate framework for assessing the quality of life of people living with HIV/AIDS and depression in Uganda. With this validation and adaptation exercise we have demonstrated that it is a transferable and suitable instrument for use in low- and middle-income country settings, with some of the aspects needing further explanations and amendments.

We recommend the use of the OxCAP-MH alongside generic HRQoL PROMs in economics evaluations until there is wider use of the OxCAP-MH within populations and in various settings, or in settings where comparison across disease conditions is sought. The OxCAP-MH will be used as a complementary PROM to the generic EQ-5D-5L in the economic evaluation of the HIV + D intervention for people with HIV and depression.

## Conclusion

Our study developed a Luganda version of the OxCAP-MH tool that is both culturally and linguistically appropriate for use in the Ugandan context and is feasible for the measurement of outcomes in people living with HIV with comorbid depression. The developed Luganda version of the OxCAP-MH measures broader wellbeing amongst Luganda speakers in Uganda using the capability approach, and can be used both as an alternative or as an addition to HRQoL measurement, both in mental health research and in research about people living with HIV with comorbid depression.

## Data Availability

Not Applicable.

## Abbreviations

AIDS: Acquired Immuno-Deficiency Syndrome
BDI-II: Beck Depression Inventory 2nd edition
cRCT: Cluster Randomised Controlled Trial
DD: Depressive Disorders
DHO: District Health Officer
EQ 5D: EuroQol Five Dimension questionnaire
HIV: Human Immune Virus
HRQoL: Health-Related Quality of Life
IA: Interviewer-Administered
MoH: Ministry of Health
OxCAP-MH: Oxford CAPabilities Questionnaire for Mental Health
PLHIVs: People Living With HIV/AIDS
PROM: Patient-Reported Outcome Measure
PSI: Population Service International
SA: Self-Administered
SF-12: 12-item Short Form survey
SF-36: 36-item Short Form survey
SF-6: 6-item Short Form survey
TCT: Translation Coordination Team
TLV: Translation and Linguistic Validation
UK: United Kingdom
